# Effects of cervical rotatory manipulation on the cervical spinal cord complex with ossification of the posterior longitudinal ligament in the vertebral canal: A finite element study

**DOI:** 10.3389/fbioe.2023.1095587

**Published:** 2023-01-13

**Authors:** Fan Xue, Hao Deng, Zujiang Chen, Han Yang, Yikai Li, Shiguo Yuan, Nansheng Zheng, Meixiong Chen

**Affiliations:** ^1^ School of Traditional Chinese Medicine, Southern Medical University, Guangzhou, Guangdong, China; ^2^ Department of Orthopaedics, Jiashan Hospital of Traditional Chinese Medicine, Jiaxing, Zhejiang, China; ^3^ Department of Orthopaedics, Hainan Traditional Chinese Medicine Hospital, Haikou, Hainan, China

**Keywords:** ossification of the posterior longitudinal ligament, cervical rotatory manipulation, spinal cord, dura, nerve roots, finite element analysis

## Abstract

**Background:** There are few studies focusing on biomechanism of spinal cord injury according to the ossification of the posterior longitudinal ligament (OPLL) during cervical rotatory manipulation (CRM). This study aimed to explore the biomechanical effects of CRM on the spinal cord, dura matter and nerve roots with OPLL in the cervical vertebral canal.

**Methods:** Three validated FE models of the craniocervical spine and spinal cord complex were constructed by adding mild, moderate, and severe OPLL to the healthy FE model, respectively. We simulated the static compression of the spinal cord by OPLL and the dynamic compression during CRM in the flexion position. The stress distribution of the spinal cord complex was investigated.

**Results:** The cervical spinal cord experienced higher von Mises stress under static compression by the severe OPLL. A higher von Mises stress was observed on the spinal cord in the moderate and severe OPLL models during CRM. The dura matter and nerve roots had a higher von Mises stress in all three models during CRM.

**Conclusion:** The results show a high risk in performing CRM in the flexion position on patients with OPLL, in that different occupying ratios in the vertebral canal due to OPLL could significantly increase the stress on the spinal cord complex.

## Introduction

Mechanical neck pain is prevalent in the general population, which can cause considerable disability and health expenditure (Martin et al., 2008; Shetty et al., 2022). Cervical Spine Manipulation (CSM) is considered an effective treatment for nonspecific mechanical neck pain (Cassidy et al., 2012). Cervical Rotatory Manipulation (CRM), one of the CSM technologies, is widely used for treating mechanical neck pain in China, and was developed by Feng, (2002). It was reported that, in China, cerebrovascular accidents, spinal cord injury and nerve root injury are the three most common adverse events after CRM (Wang et al., 2012).

Therefore, in order to prevent the occurrence of adverse events after CRM, it is necessary to explore the mechanism of CRM on the blood vessels, spinal cord and nerve roots. Our previous studies of cerebrovascular accidents after CRM showed that CRM can significantly affect the carotid atherosclerotic plaques and vascular haemodynamic properties (Zhang et al., 2017; Qi et al., 2019).

However, little information is available concerning the mechanism of spinal cord injury and nerve root injury after CRM. Thus, there is a need to investigate when and how it happened. In our previous research, we constructed a 3-dimensional craniocervical finite element (FE) model based on a healthy male volunteer (Xue et al., 2021). Through the simulation of the three different positions (flexion, extension and neutral positions) of CRM, we found that the spinal cord experienced lower von Mises stress and wider free space in the vertebral canal after CRM in the flexion position on a healthy individual, which indicated that performing CRM in the flexion position was less likely to injure the spinal cord than in the extension and neutral positions. Nevertheless, CRM in the flexion position still showed some risks of spinal cord injury when patients with posterior vertebral space-occupying lesions such as ossification of the posterior longitudinal ligament (OPLL), especially in the C5 and C6 segments, in that the cervical enlargement begins in these segments and the cervical spinal cord was anteriorly positioned during the CRM in the flexion position.

To our knowledge, the specific biomechanical effects of CRM on the cervical spinal cord complex with OPLL have not been studied before. OPLL is one of the major contributors to myelopathy (Sun et al., 2020), whose morbidity is approximately 3.0% in Asian countries (Boody et al., 2019). Therefore, the effects of CRM on the cervical spinal cord, dura matter and nerve roots with mild, moderate, and severe OPLL were investigated quantitatively in this study. The finite element analysis method was used in this study.

In 1973, Belyschko et al. applied the FE technique to analyze the biomechanical properties of the spine and developed the first FE model of the spine (Belytschko et al., 1973). Subsequently, many researchers developed their own FE models of the cervical spine and studied the biomechanical properties of the extraspinal structures such as the cervical vertebral bodies, intervertebral discs and ligaments (Brolin and Halldin, 2004; Womack et al., 2011; Wang et al., 2016). In these studies, the findings contributed to a more accurate FE model of the cervical spine but did not involve vertebral canal contents. Some other studies have constructed cervical spinal cord models, which contributed to the static compression of the spinal cord (Li and Dai, 2009; Rui et al., 2020).

In this study, we constructed the craniocervical FE model with OPLL and vertebral canal contents on the basis of the previous study, and the compression of OPLL on the spinal cord was simulated under static and dynamic conditions, which is also the novelty of this study.

Consequently, we hypothesized that although CRM in the flexion position is safer than that in the extension or neutral positions, it is not suitable for all patient conditions, especially patients with vertebral canal stenosis such as OPLL. The objective of this study was (1) to explore the effects of CRM on the cervical spinal cord, dura matter and nerve roots in the flexion position when suffering from OPLL and (2) to distinguish the biomechanical differences of the spinal cord complex between mild, moderate, and severe OPLL.

## Materials and methods

### Model construction

A healthy three-dimensional (3D) FE model of the human craniocervical spine and spinal cord complex (spinal cord, pia matter, dura matter, cerebrospinal fluid (CSF), nerve roots, nerve rootlets and denticulate ligaments (DLs)) was constructed based on our previous study. The geometry of the cervical spine was obtained from computed tomography (CT) images at .625 mm intervals for a healthy 32-year-old male volunteer with a height of 175 cm. Volunteer’s informed consent and Chinese Ethics Committee approval were obtained before initiating this study. Mimics 19.0 software (Materialise, Leuven, Belgium) and Geomagic Wrap 2017 software (Raindrop, Marble Hill, New York) were used to obtain a high-quality nonuniform rational B-splines (NURBS) surface model of the basilaris cranii and C1∼C7 vertebral bodies. The spinal cord complex, including the spinal cord, pia matter, dura matter, CSF, nerve roots, nerve rootlets and DLs, was constructed using Solidworks 2017 software (Dassault Systems SA, Waltham, Massachusetts) based on published anatomical data (Kameyama et al., 1996; Ko et al., 2004). The whole model was constructed in the Cartesian coordinate system, where the *y*-axis was the sagittal direction, and the *z*-axis was the axial direction of the model.

Specifically, we used Mimics software to extract the original 3D geometry based on the CT images obtained before. Gray value was used as a reference tool to extract the bones into a mask. We used 226∼3071 Hounsfield unit (HU) as the threshold range for the gray value. The “Region Growing” tool was used to split the mask into a separate object. After that, the “Split Mask” tool can allow us to obtain the separate cervical vertebrae bodies and basilaris cranii. The “Edit Mask” tool was used to fill the holes in a mask.

The STL format files, which contained the original 3D geometry of basilaris cranii and C1∼C7 vertebral bodies, were imported into Geomagic Wrap software. The “Remesh” command was used to retriangulate a polygon mesh, which can be beneficial to the subsequent operations. The target edge length was set to .35 mm. The “Defeature” command can help to remove features that are not important, and the “Relax” command was used to smooth the bones. After that, we used the “Contours”, “Patches”, “Grids” and “Surfaces” modules respectively to generate a NURBS surface. Also, we applied a uniform offset of 0.4 mm to generate the cancellous bone model. According to Panjabi et al., the thickness of cortical bone varies in different parts of the cervical spine in a healthy individual (Panjabi et al., 2001a; Panjabi et al., 2001b). However, since the cervical vertebrae bodies and the basilaris cranii were not our focus in this study, the offset was uniformly set to .4 mm for the convenience of FE analysis (Mo et al., 2014). The NURBS surfaces of basilaris cranii and C1∼C7 vertebral bodies consisted of 17,044 patches and 1665240 triangles.

Since the structures such as intervertebral discs, zygapophysial cartilage, nerve roots and spinal canal contents cannot be distinguished clearly by the grey value, Solidworks software was used to construct these objects based on their anatomical positions (Kameyama et al., 1996; Ko et al., 2004). In terms of the zygapophysial cartilage, we sketched out the superior and inferior surfaces separately in the sketch module. The guide curves can assist in forming the lateral aspect of the cartilage. The “Loft” tool was used to generate the entity of zygapophysial cartilage. All the cartilage was inserted into the space of the zygapophysial joints. In the same way, we constructed the endplate, annulus fibrosus and nucleus pulposus to form the intervertebral disc. The thickness of each endplate was .4 mm (Mo et al., 2014). The adult nucleus pulpous makes up 35%–50% of the intervertebral disc (Bishop, 1992), and we assumed that the volume of the nucleus pulpous made up approximately 40% of the entire disc in this study (Denozière and Ku, 2006). The ligaments included in this study were the anterior longitudinal ligament (ALL), posterior longitudinal ligament (PLL), ligamentum flavum (LF), capsular ligament (CL), transverse ligament (TL), alar ligament (AL), apical ligament of the odontoid process (APL), intertransverse ligament (IL), supraspinous ligament (SSL) and interspinous ligament (ISL). These ligaments were realized by the “3D sketch” tool in Solidworks. We improved Khuyagbaatar’s methods to construct our spinal cord complex. It was reported that the ratio of the cross-sectional area of each spinal cord segment to that of the C3 segment was similar in different populations (Kameyama et al., 1996). Meanwhile, Ko et al. performed detailed measurements of the sagittal diameter, transverse diameter, cross-sectional area, and ratio of gray to white matter (Ko et al., 2004). Therefore, the model of the cervical spinal cord in this study was constructed exactly according to the data from the literature. Also, we included the pia matter in our model that can protect the gray and white matter. The pia matter was tied to the outer surface of the white matter, while the dura matter was placed approximately 1.5–4.0 mm from the pia matter. CSF filled the area between the pia matter and the dura matter. The ventral and dorsal nerve rootlets (with seven rootlets for each side) extended anterolaterally to form the nerve root (Cargill et al., 1998; Chang et al., 2006). The DLs were constructed based on a published anatomical study (Katarzyna et al., 2018).

**FIGURE 1:**
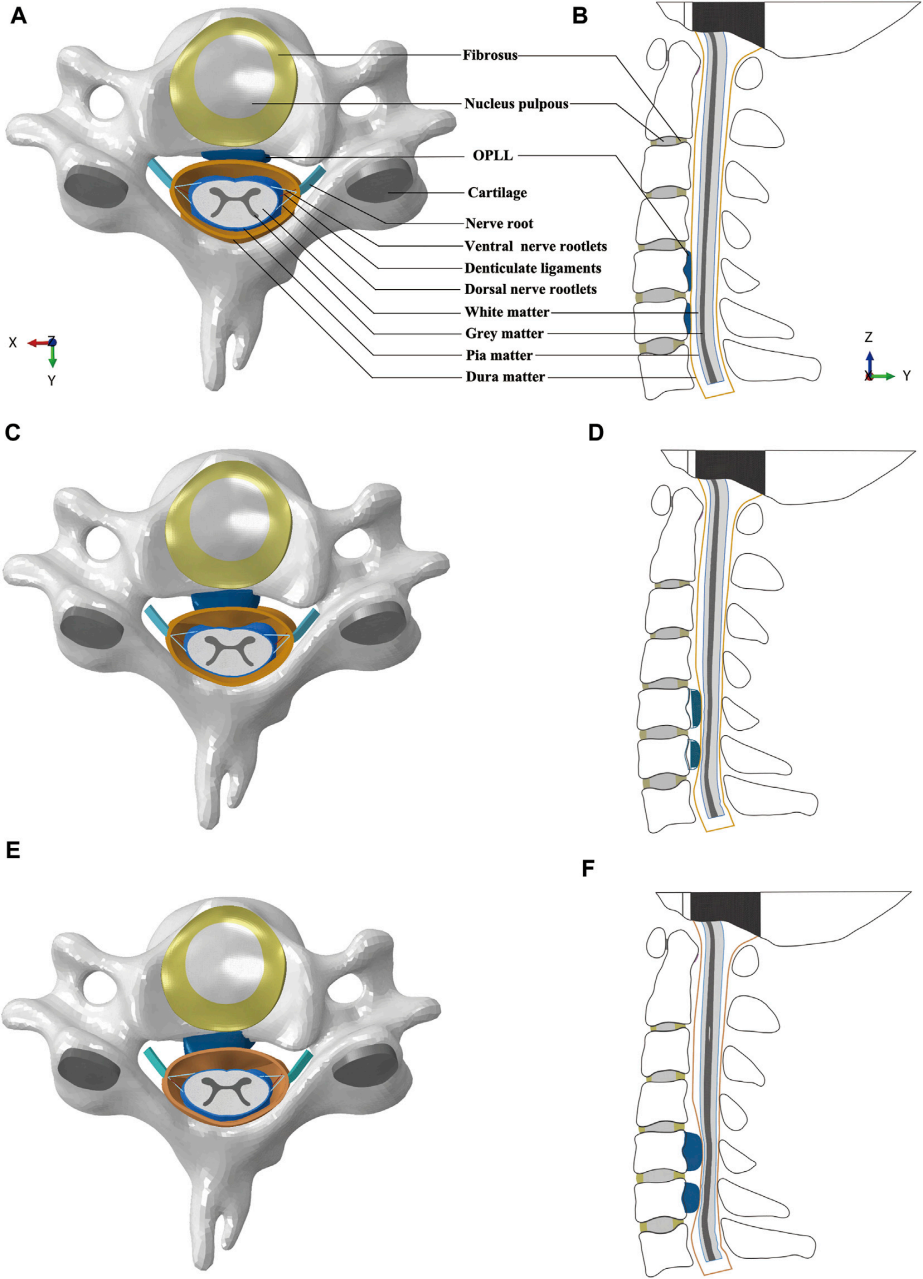
Intact FE models of cervical spine and spinal cord complex: **(A)** Transverse view of C5 with mild OPLL. **(B)** Sagittal view of the FE model with mild OPLL at the C5 and C6 segments. **(C)** Transverse view of C5 with moderate OPLL. **(D)** Sagittal view of the FE model with moderate OPLL at the C5 and C6 segments. **(E)** Transverse view of C5 with severe OPLL. **(F)** Sagittal view of the FE model with severe OPLL at the C5 and C6 segments. (FE, finite element; OPLL, ossification of the posterior longitudinal ligament).

Three different models were developed by adding mild, moderate, and severe OPLL to the healthy FE model. The OPLL was classified as mild, moderate, or severe based on the occupying ratio of spinal canal stenosis, which was defined as the ratio of OPLL thickness to the sagittal diameter of the cervical vertebral canal (Matsunaga and Sakou, 2012; Khuyagbaatar et al., 2015). The occupying ratios of mild, moderate, and severe OPLL in the vertebral canal were considered to be 20%, 40% and 60% according to radiographic findings, respectively (Matsunaga et al., 2015) (Figure 1). In this study, we constructed the segmental plateau-shaped OPLL in consideration of its high prevalence based on a commonly used classification system by the Investigation Committee for Ossification of the Spinal Ligaments (Tetreault et al., 2018). Our previous study showed that the free space of the spinal cord at the C5/6 segment after CRM in the flexion position was relatively narrow. Accordingly, OPLL at the C5 and C6 segments was constructed in this study (Figure 1).

### Model pre-processing

ABAQUS 2020 (Simulia/Dassault Systèmes, Vélizy-Villacoublay, France) was used to complete the preprocessing and analysis. Specifically, the OPLL was modelled as a rigid body (Khuyagbaatar et al., 2018). The white and grey matter were modelled as hexahedral solid elements that were nonlinear and hyperelastic. Their stress-strain curves were obtained from an *in vitro* experiment (Ichihara et al., 2001). The dura matter and pia matter were also modelled as hexahedral solid elements with thicknesses of .1 mm and .4 mm, respectively (Holsheimer et al., 1994; Ozawa et al., 2004); their material properties were linear and elastic (Persson et al., 2010; Stoner et al., 2020; Jannesar et al., 2021). The nerve rootlets and DLs were modelled as 3D truss elements; their material properties were nonlinear and elastic-plastic, and their stress-strain curves were obtained from published biomechanical studies (Singh et al., 2006; Polak et al., 2014). The volume between the dura matter and pia matter was filled with CSF, and the interaction between the CSF and the solid bodies was investigated *via* the smoothed-particle-hydrodynamics (SPH) analysis method using ABAQUS 2020. CSF was modelled as hexahedral solid elements and was converted to mass particles at the beginning of the analysis. The material properties and viscosity of the CSF demonstrated characteristics of a Newtonian fluid (Panzer et al., 2012; Jannesar et al., 2021). The material properties used in our model are summarized in Table 1.

**TABLE 1:** Material properties and element types used in the current model.

Component	Element type	Material type	Material parameters
Cortical	Solid, C3D8R^a^	Elastic	E^c^ = 12,000 MPa υ^d^ = .29 ρ^e^ = 1.83E-09 tonne/mm^3^
Cancellous	Solid, C3D8R	Elastic	E = 450 MPa υ = .29 ρ = 1.00E-09 tonne/mm^3^
OPLL	Solid, C3D8R	Rigid body	ρ = 1.83E-09 tonne/mm^3^
Cartilage	Solid, C3D8R	Elastic	E = 10 MPa υ = .3 ρ = 1.20E-09 tonne/mm^3^
Grey matter	Solid, C3D8R	Hyperelastic	μ^f^ = 4.1 kPa α^g^ = 14.7 ρ = 1.05E-09 tonne/mm^3^
White matter	Solid, C3D8R	Hyperelastic	μ = 4.0 kPa α = 12.5 ρ = 1.05E-09 tonne/mm^3^
Pia matter	Solid, C3D8R	Elastic	E = 39.3 MPa υ = .3 ρ = 1.13E-09 tonne/mm^3^
Dura matter	Solid, C3D8R	Elastic	E = 80 MPa υ = .49 ρ = 1.174E-09 tonne/mm^3^
DLs	3D truss, T3D2^b^	Elasticplastic	Stress-strain curve ρ = 1.13E-09 tonne/mm^3^
Nerve rootlet	3D truss, T3D2	Elasticplastic	Stress-strain curve ρ = 1.13E-09 tonne/mm^3^
Nerve root	Solid, C3D8R	Elastic	E = 1.3 MPa υ = .3 ρ = 1.00E-09 tonne/mm^3^
CSF	Solid	Mie-Grüneisen equations of state, Newtonian fluid	co^h^ = 1381700 mm/s
			s^i^ = 1.979
			Γ0^j^ = .11
			μ^k^ = .0008 Pa s
			ρ = 1.007E-09 tonne/mm^3^

^a^ An 8-node linear brick, reduced integration, hourglass control.

^b^ A 2-node linear 3D truss.

^c^ Elasticity modulus.

^d^ Poisson’s ratio.

^e^ Density.

^f^ Shear modulus.

^g^ Strain hardening index.

^h^ Sound velocity.

^i^ A constant defining the linear relationship between the shock velocity and the particle velocity.

^j^ Mie-Grüneisen ratio (A material constant).

^k^ Viscosity.

OPLL, ossification of the posterior longitudinal ligament; DLs, denticulate ligaments; CSF, cerebrospinal fluid.

To ensure that the findings of our study were accurate, two simulations were performed in ABAQUS 2020 to validate our FE model. First, a normal force of .8N was applied to the ventral surface of the middle segment of the spinal cord when the cephalic, caudal and dorsal sides of the spinal cord are fixed, and the corresponding displacement was calculated. The force-displacement relationship was close to the results of the published *in vitro* study (Hung et al., 1982). This validation indicated the accuracy of the mechanical properties of the spinal cord model used in this study. Second, an axial rotation of ± 20° around the *x*-axis was simulated to allow cervical flexion and extension. The anterior-posterior, left-right and superior-inferior displacement of the C3∼C7 spinal cord were calculated, and the results matched the *in vivo* experimental data performed by Stoner et al. well (Stoner et al., 2019). This validation indicated the accuracy of spinal cord kinematics in the overall model. As a similar validation had been performed in the previous study, images of the model validation results were not shown here. Also, we performed a convergence check to ensure that the mesh density was acceptable. The convergence check was performed on the mesh of the spinal cord, dura matter and nerve roots.

### OPLL compression and CRM simulation

**FIGURE 2:**
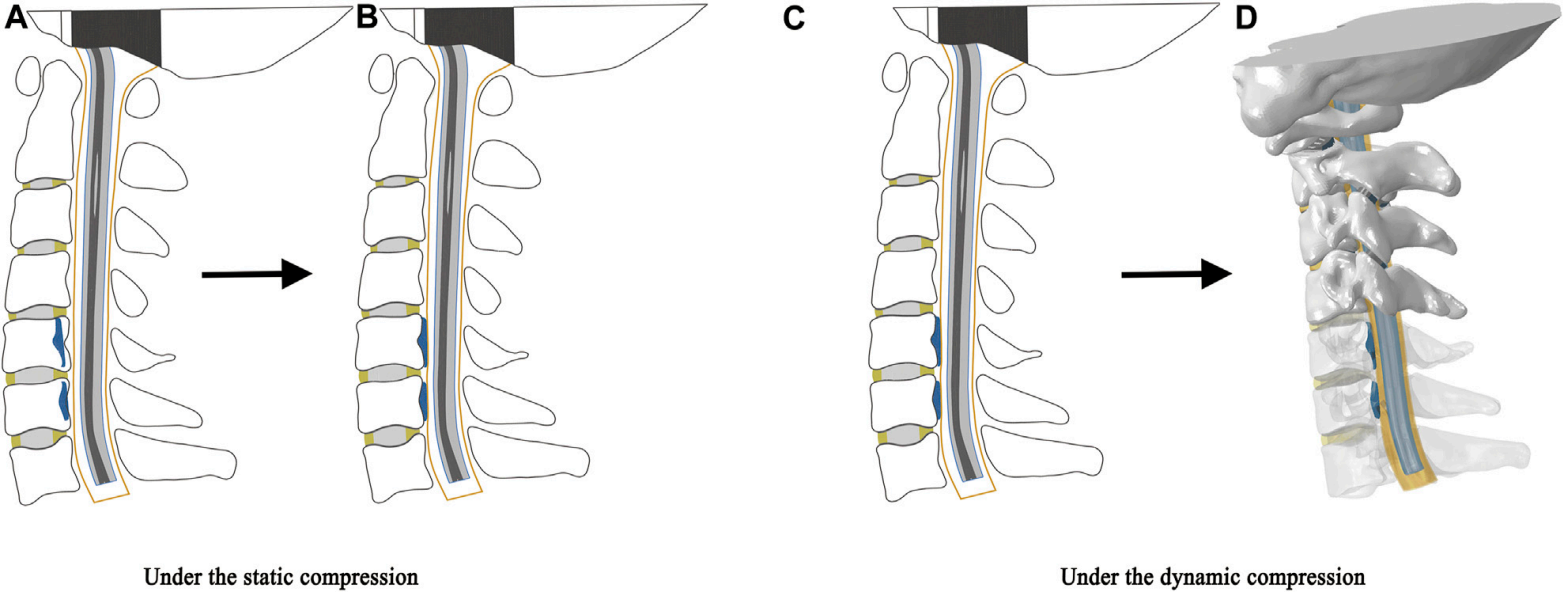
Schematic illustration of static and dynamic compression. The static compression: **(A)** The OPLL model was initially placed inside the vertebral bodies. **(B)** The OPLL model moved in the direction perpendicular to the anterior surface of the dura matter to compress the cervical spinal cord when all the parts were fixed except for the OPLL. The dynamic compression: **(C)** The OPLL model was tied to the posterior surface of the vertebral body when the static compression was completed. **(D)** CRM in the flexion position was performed with the OPLL compression on the spinal cord. (CRM, cervical rotatory manipulation).

This study simulated the compression of OPLL on the spinal cord under static and dynamic conditions (Figure 2). Briefly, the compression of the spinal cord by the OPLL before CRM is called static compression, while the compression during CRM is called dynamic compression. Under the static condition, the OPLL model was initially placed inside the vertebral bodies (Figure 2A) and then moved by the occupying ratio (20%, 40%, or 60%) in the direction perpendicular to the anterior surface of the dura matter in order to compress the cervical spinal cord when all the parts were fixed except for the OPLL (Figure 2B). Compression under the dynamic condition continued on the basis of static compression. That is, the OPLL model was tied to the posterior surface of the vertebral body when the static compression was completed (Figure 2C) and then the rotation was performed on the model (CRM in the flexion position was performed with mild, moderate, and severe OPLL compression on the spinal cord) (Figure 2D).

A sequence of loading steps to simulate CRM in the flexion position followed our previous research (Mei and Li, 2013) and published FE studies (Zhen et al., 2017). In order to save calculation time, we shortened the time period in the Explicit analysis. We applied a boundary condition (rotation) at the rigid body reference point (center of mass) of the basilaris cranii to simulate the CRM. In detail, first, a 15° rotation around the *x*-axis was applied to the basilaris cranii to simulate craniocervical flexion. Second, in order to enter the passive end range of motion, a −60° rotation around the *z*-axis was applied to simulate craniocervical axial rotation in the right direction. In clinical practice, flexion and axial rotation are often performed simultaneously. Therefore, in this study, 15° of flexion and 60° of axial rotation occurred simultaneously within .03 s. Finally, a continuous axial rotation of 4° around the *z*-axis was applied within 5E-04 s (approximately 4 times the rotation rate of the previous step) to enter the paraphysiological movement zone (Herzog, 2010).

### Data analysis

The von Mises stress on the spinal cord, dura matter, and nerve roots was measured under various OPLL occupying ratios. Specifically, we measured the maximum von Mises stress on the spinal cord and dura matter under rightward 4° *z*-axis rotation during CRM. One maximum stress data was measured for each increment during this process. In addition, we measured the von Mises stress at each integration point of the C1∼C8 nerve roots after CRM in the flexion position. SPSS 21.0 statistical software (IBM Corporation, Armonk, New York,US) was used for the statistical analysis. The measured data were expressed in the form of the average and standard deviation, ( $\bar{x}$ ± SD). Statistically, significant differences among the data between the different OPLL groups (mild, moderate, and severe) were determined by a one-way Analysis of Variance in conjunction with a multiple comparison test (Games-Howell). Because the data distributions were of heterogeneous variances, Welch’s test was performed. Differences were considered significant when the *P* values were < .05.

## Results

### Stress distribution on the spinal cord

Under static OPLL compression, the spinal cord in the severe OPLL model experienced higher von Mises stress compared with that in the mild and moderate OPLL. The maximum von Mises stress of the spinal cord under static OPLL compression was lower than that under dynamic OPLL compression.

**FIGURE 3:**
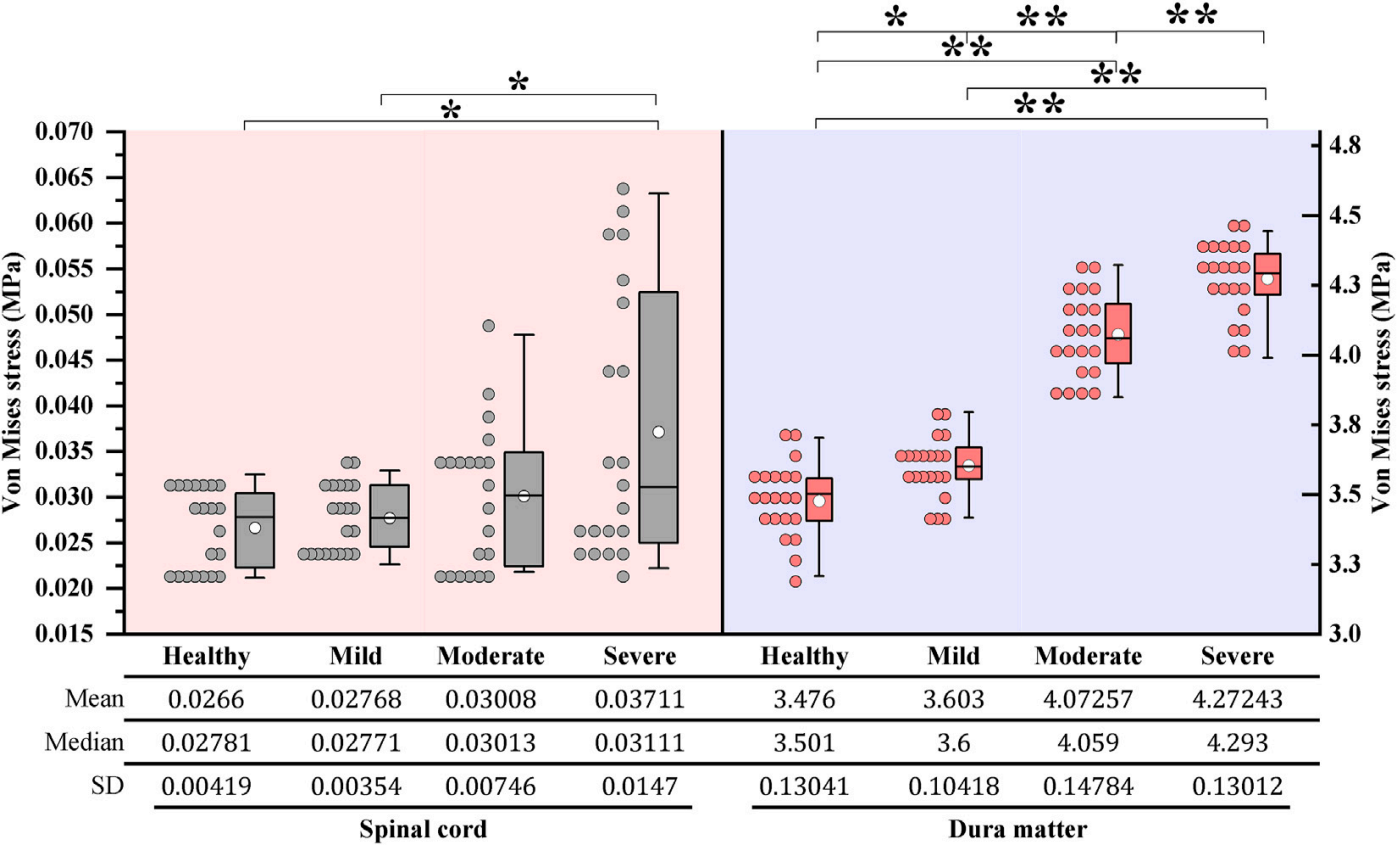
Boxplots of the von Mises stress of the cervical spinal cord and dura matter during CRM: One maximum stress data was measured for each increment under 4° *z*-axis rotation during CRM. On each box, the central thick line indicates the median, the white circle marker indicates the mean, and the top and bottom edges indicate the 75th and 25th percentiles, respectively. The whiskers extending to the maximum and minimum values do not consider outliers. *p < .05, **p < .001. (CRM, cervical rotatory manipulation).

**FIGURE 4:**
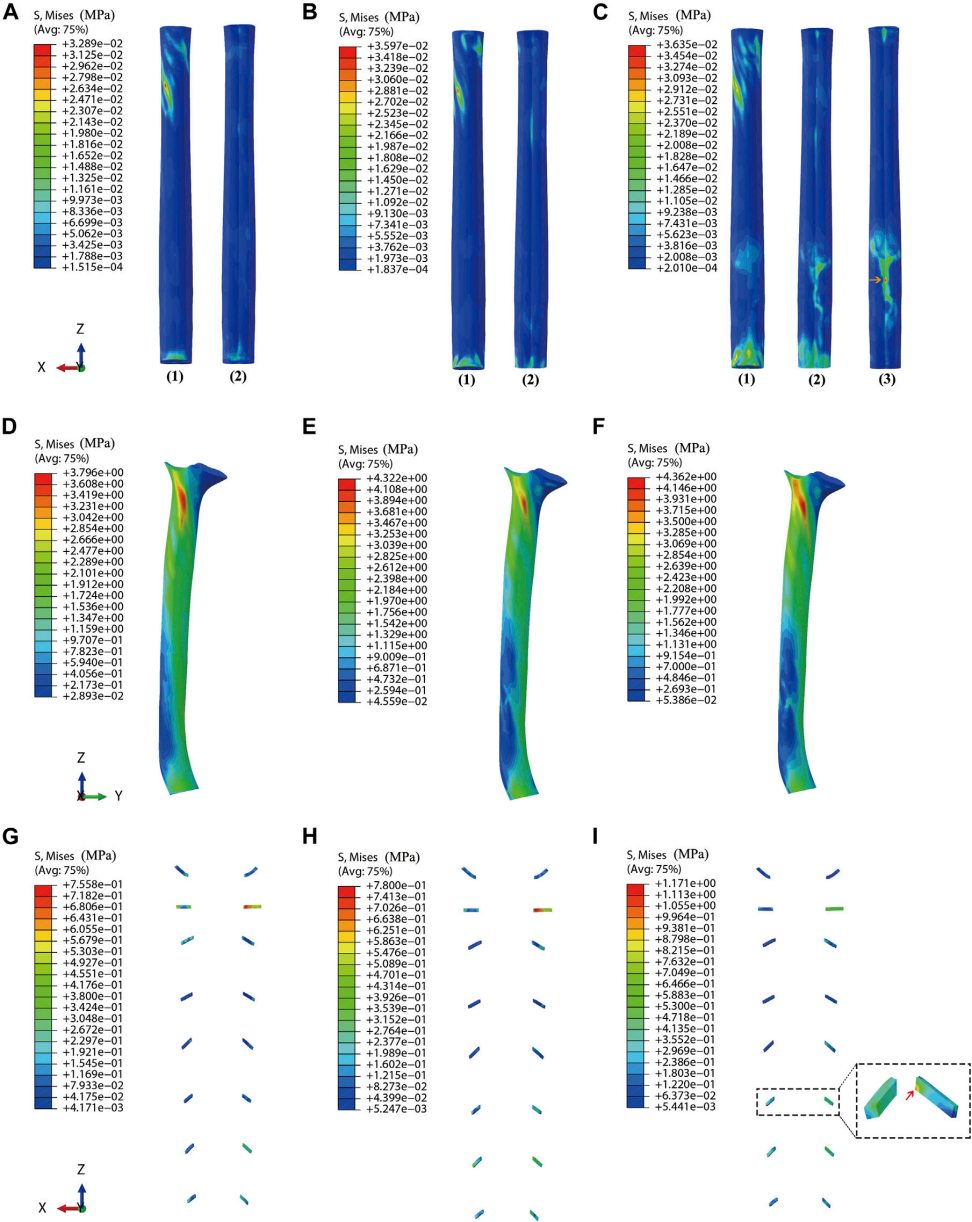
Stress distribution on the spinal cord, dura matter, and nerve roots **(A)** Stress distribution on the spinal cord for the model with mild OPLL. **(B)** Stress distribution on the spinal cord for the model with moderate OPLL. **(C)** Stress distribution on the spinal cord for the model with severe OPLL. (1) Dorsal surface of the spinal cord. (2) Ventral surface of the spinal cord. (3) The maximum von Mises stress at the C5/6 segment (compression level of the OPLL) during the first half of 4° *z*-axis rotation (orange arrow). **(D)** Stress distribution on the dura matter for the model with mild OPLL. **(E)** Stress distribution on the dura matter for the model with moderate OPLL. **(F)** Stress distribution on the dura matter for the model with severe OPLL. **(G)** Stress distribution on the nerve roots for the model with mild OPLL. **(H)** Stress distribution on the nerve roots for the model with moderate OPLL. **(I)** Stress distribution on the nerve roots for the model with severe OPLL, and the maximum von Mises stress was at the C6 segment (red arrow). (OPLL, ossification of the posterior longitudinal ligament).

Under dynamic OPLL compression, the average von Mises stress of the spinal cord under rightward 4° *z*-axis rotation during CRM was .0277, .0301, and .0371 MPa for the mild, moderate, and severe OPLL models, respectively. Compared with the model without OPLL, the von Mises stress increased by 4.1% (*p* = .802), 13.1% (*p* = .265) and 39.5% (*p* = .021) in the mild, moderate, and severe OPLL models, respectively (Figure 3). Although there was no significant difference between the healthy model and the moderate OPLL model throughout the whole process of the rightward 4° *z*-axis rotation, we found that the von Mises stress of the spinal cord in the moderate OPLL model was higher than that in the healthy model during the latter half of the 4° *z*-axis rotation (*p* = .009). Additionally, the maximum von Mises stress of the spinal cord during CRM was at the C1/2 segment in the mild and moderate OPLL models. Interestingly, in the severe OPLL model, the maximum von Mises stress of the spinal cord was at the C5/6 disc level, which was the compression level of the OPLL, during the first half of the 4° *z*-axis rotation (orange arrow in Figure 4), while the maximum von Mises stress was at the C1/2 segment during the latter half.

### Stress distribution on the dura matter

To evaluate the situation where the OPLL had no contact with the spinal cord but did contact the dura matter, the stress distribution of the dura matter was also observed. Under static OPLL compression, the maximum von Mises stress of the dura matter was much lower than that under the dynamic condition.

Under dynamic OPLL compression, the average von Mises stress of the dura matter under 4° *z*-axis rotation during CRM was 3.6030, 4.0726, and 4.2724 MPa for the mild, moderate, and severe OPLL models, respectively. The von Mises stress of the dura matter in the mild, moderate, and severe OPLL models was 3.7% (*P* = .006), 17.2% (*P* < .001) and 22.9% (*P* < .001) higher than that without OPLL, respectively (Figure 3). Furthermore, the maximum von Mises stress of the dura matter during CRM was at the C1/2 segment in both the OPLL and without OPLL models (Figure 4).

### Stress distribution on the nerve roots

The OPLL may have a side effect on the nerve roots after CRM, which increased the von Mises stress of the nerve roots around the compression area (Figure 4). Under static OPLL compression, the maximum von Mises stress of the nerve roots was located at the C6 segment, and the value was much lower than that under the dynamic condition.

Under dynamic OPLL compression, the von Mises stress of the nerve roots increased by 22.1% (*P* < .001) at the C6 segment in the mild OPLL model compared with that of the without-OPLL model (Table 2). With the increase of OPLL occupying ratios, more nerve roots segments experienced the increased von Mises stress. That is, the von Mises stress of the nerve roots in the moderate OPLL model was 9.6% (*P* = .036), 109.5% (*P* < .001), 53.6% (*P* < .001), and 30.3% (*P* < .001) higher than that without OPLL at the C5 ∼ C8 segments, respectively (Table 2). For the OPLL model, a higher von Mises stress of the nerve roots was found at the C1 and C5 ∼ C8 segments (*P* < .001), which increased by 16.3%, 38.1%, 248.4%, 81.1%, and 15.2% compared with that of the C1 and C5∼C8 segments in the model without OPLL, respectively (Table 2). Additionally, the maximum von Mises stress of the nerve roots during CRM was at the C2 segment in the mild and moderate OPLL models, while it was at the C6 segment in the severe OPLL model (red arrow in Figure 4).

**TABLE 2:** Von Mises stress of the nerve roots (units:MPa).

Occupying ratios of the OPLL (%)	C1	C2	C3	C4	C5	C6	C7	C8
0	.1173 ± .0381	.4492 ± .1914	.1450 ± .0786	.0636 ± .0429	.0672 ± .0381	.0793 ± .0337	.1878 ± .0666	.1580 ± .0585
20	.1141 ± .0581	.4394 ± .1938	.1485 ± .0798	.0550 ± .0479	.0730 ± .0507	.0968 ± .0379**	.1896 ± .0784	.1516 ± .0494
40	.1219 ± .0422	.3945 ± .2563	.1007 ± .0725	.0610 ± .0497	.0736 ± .0410*	.1661 ± .0745**	.2884 ± .0653**	.2058 ± .0691**
60	.1365 ± .0442**	.4028 ± .2510	.1080 ± .0871	.0691 ± .0442	.0928 ± .0925**	.2763 ± .1061**	.3400 ± .0619**	.1820 ± .0513**

Data expressed as $\bar{x}$ ± SD.

* denotes *p* < .05 significance vs the model without OPLL.

** denotes *p* < .001 significance vs the model without OPLL.

OPLL, ossification of the posterior longitudinal ligament.

## Discussion

The novel element of this study is two-fold. On the one hand, the research contents are new. That is, the specific biomechanical effects of CRM on the cervical spinal cord complex with OPLL have not been studied before, and the compression of OPLL on the spinal cord was simulated under static and dynamic conditions. On the other hand, the FE model is new. That is, we reconstructed an intact FE model, including the C1 ∼ C7 vertebral bodies, basilaris cranii, intervertebral disc, zygapophysial cartilage, nerve root, vertebral canal contents (spinal cord, pia matter, dura matter, cerebrospinal fluid and denticulate ligaments) and ossified posterior longitudinal ligament. It is believed that there is stretch tension in the kyphotic dura that generates a forward force to cause the dura matter to approach the anterior wall of the vertebral canal when craniocervical flexion occurs (Yuan et al., 1998), which is in line with our previous research (Xue et al., 2021). Accordingly, CRM in the flexion position was simulated in this study to explore the biomechanical characteristics of the spinal cord complex when suffering from OPLL and to distinguish the biomechanical difference of the spinal cord complex between mild, moderate, and severe OPLL. Three validated FE models of the craniocervical spine and spinal cord complex were constructed by adding mild, moderate, and severe OPLL to the healthy FE model. The findings of this study can provide data support for the rational application of CRM. It also explains why adverse events like spinal cord injury and nerve root injury sometimes occurred after CRM, and how such adverse events can be avoided. Different occupying ratios due to OPLL exhibited different biomechanical properties, which explains the diversity of symptoms in clinics, and we should treat them differently. The details are as follows.

Stress distribution can provide critical information for exploring the injury mechanism of the spinal cord. In this study, we found that the cervical spinal cord experienced higher von Mises stress under static compression from severe OPLL, which indicated that occupying ratios in the vertebral canal due to OPLL greater than 60% under the static factor may be a risk factor for the development of myelopathy. Matsunaga et al. also previously noted that all patients in their study with >60% spinal canal stenosis due to OPLL exhibited cervical myelopathy (Matsunaga et al., 2015).

Regarding the dynamic compression, we simulated CRM in the flexion position. Different occupying ratios in the vertebral canal due to OPLL led to different stresses on the spinal cord, dura matter, and nerve roots. A higher von Mises stress of the spinal cord was found in the model with severe OPLL during CRM, which agrees with the results of Kim et al., who reported that a 60% compression in OPLL can be a threshold for neurologic symptoms (Kim et al., 2013). Meanwhile, a significant difference between the von Mises stress of the spinal cord in the healthy model and the moderate OPLL model was found during the latter half of CRM, which demonstrated that, under the same degrees of axial rotation, patients with ≥40% occupying ratios due to OPLL were likely to experience spinal cord injury under CRM. Therefore, the dynamic factors should be considered in the pathomechanism of cervical myelopathy. Other researchers previously reported that dynamic factors appear to be more important for the development of myelopathy in patients with less than 60% spinal canal stenosis (Matsunaga and Sakou, 2012; Matsunaga et al., 2015; Nishida et al., 2015 Boody et al., 2019), which agrees with the results in this study. Lee et al. utilized sensory-evoked potentials to show that occupying ratios at 56.3% and 87.4% led to rat spinal cord dysfunction, while no significant cord dysfunction occurred at 23.7% compression (Lee et al., 2011). Thus, it can be concluded that elevated spinal cord stress is related to the neurologic symptoms in patients. Here, from a biomechanical viewpoint, a 60% compression due to OPLL under the static factor and 40% compression due to OPLL under the dynamic factor may be regarded as thresholds for symptoms.

The maximum von Mises stress of the spinal cord occurred at the C1/2 segment during CRM in the mild and moderate OPLL models, which was attributed to the ranges of axial rotation at the atlantoaxial joint (Cramer and Darby, 2005). The atlantoaxial joint allows for axial rotation of 50% at the cervical spine, which is consistent with results from Cramer et al., who reported that the range of the unilateral axial rotation at the atlantoaxial joint is 28 ∼ 40° (Cramer and Darby, 2005). Moreover, from the orange arrow in Figure 4, we can see that in the severe OPLL model, the maximum von Mises stress of the spinal cord was at the C5/6 segment (compression area due to OPLL) during the first half of CRM, while it was at the C1/2 segment during the latter half of CRM, which is consistent with the results of Koyanagi et al., who reported that spinal cord injury always occurred at disc levels adjacent to segmental-type OPLL (Koyanagi et al., 2003). Therefore, patients with 40% occupying ratios due to OPLL were likely to experience spinal cord injury at the C1/2 segment instead of the compression area during CRM, while both the C1/2 and C5/6 segments of the spinal cord were likely to be injured in patients with spinal canal stenosis greater than 60%. The higher up in the spine that the spinal cord injury occurs, the more severe the potential outcome. Clinically, injuries to the spinal cord at the C1/2 segment are considered to be the most severe, as they can cause full paralysis or death, depending on the classification and severity of the injury. Injuries to the C5/6 segment of the spinal cord can lead to tetraplegia. Although they usually have better outcomes than higher cervical spinal cord injuries, they are still considered very severe because of the significant psychologic concern for the patients (Cramer and Darby, 2005).

Increased stress in the dura matter due to compression, is clinically correlated with neckache or headaches. In recent years, many anatomic and MRI studies have confirmed the existence of a myodural bridge (connection between the musculoskeletal system and the dura mater), which is considered to play an important role in the etiology of neckache and headache (Kahkeshani and Ward, 2012; Palomeque-del-Cerro et al., 2017). The dura matter is innervated by the C1∼C3 spinal nerves and is proved to contain large numbers of mast cells and sensory nerves with substance P, both markers of pain sensitivity (Kahkeshani and Ward, 2012). In this study, the von Mises stress of the dura matter in the mild, moderate, and severe OPLL models was significantly higher than that in the model without OPLL, which demonstrated that patients with OPLL may aggravate neckache or headache after CRM; this has been previously reported of nerve injury after CRM (Puentedura et al., 2012). Similarly, in this study, from Figure 4, we noticed that the von Mises stress of the nerve roots increased adjacent to the OPLL compression area, suggesting that it was likely to aggravate radicular pain or neurological dysfunction in patients with OPLL after CRM.

Clinically, CRM in the flexion position is widely used in China, in that it is considered to be a relatively safe position (Feng, 2002); however, it still shows high risk in performing CRM on patients with OPLL (Puentedura et al., 2012). Specifically, patients with mild OPLL may aggravate neckache, radicular pain, or headache after CRM. Patients with moderate or severe OPLL may also suffer from spinal cord injury or myelopathy after CRM. Thus, CRM should be used with caution if there is vertebral canal stenosis such as OPLL, and both static and dynamic factors should be considered in the development of OPLL. Generally, patients with OPLL exhibit restricted cervical range of motion. However, patients with OPLL exhibiting no restricted cervical range of motion are susceptible to cervical myelopathy caused by dynamic factors (Matsunaga et al., 2004). A comprehensive evaluation of medical history, clinical symptoms, physical examination, and radiographic examination is necessary before CRM (Vautravers and Maigne, 2000).

There were some limitations in this study. First, only the segmental plateau-shaped OPLL was constructed. It is necessary to investigate various types of OPLL in future studies because the stress on the spinal cord may be more affected by the shape of the OPLL, where a more angular shape may lead to higher stress. In addition, the OPLL models were constructed based on a healthy model in consideration of the principles of controlling variables and the CRM simulations were simplified, which may not be completely consistent with the actual situation but is helpful in terms of comparability and reproducibility. Third, only the stress distribution was investigated, and we used the von Mises stress failure criterion for human tissues. Other causal factors that can contribute to spinal cord injury, including displacement or ischemia of the spinal cord, were not taken into account.

In conclusion, the present study can quantitatively predict biomechanical characteristics of the spinal cord complex and provides valuable information for understanding the correlation between the neurologic symptoms and mechanical stress on the spinal cord complex due to OPLL. The results suggested that certain occupying ratios in the vertebral canal due to OPLL could significantly increase the stress of the spinal cord complex. Clinically, this indicates high risk in performing CRM in the flexion position on patients with OPLL, and caution is warranted when spinal canal stenosis exists.

## Data Availability

The raw data supporting the conclusion of this article will be made available by the authors, without undue reservation.
